# Supplementary material for: Regulation of Cancer Stem Cells and Epithelial-Mesenchymal Transition by CTNNAL1 in Lung Cancer and Glioblastoma
Source: Biomedicines. 2023 May 17;11(5):1462. doi: 10.3390/biomedicines11051462 (PMC10216818; doi:10.3390/biomedicines11051462)
Supplement: Supplementary file 1 [file biomedicines-11-01462-s001.zip › biomedicines-2353276-supplementary.pdf]

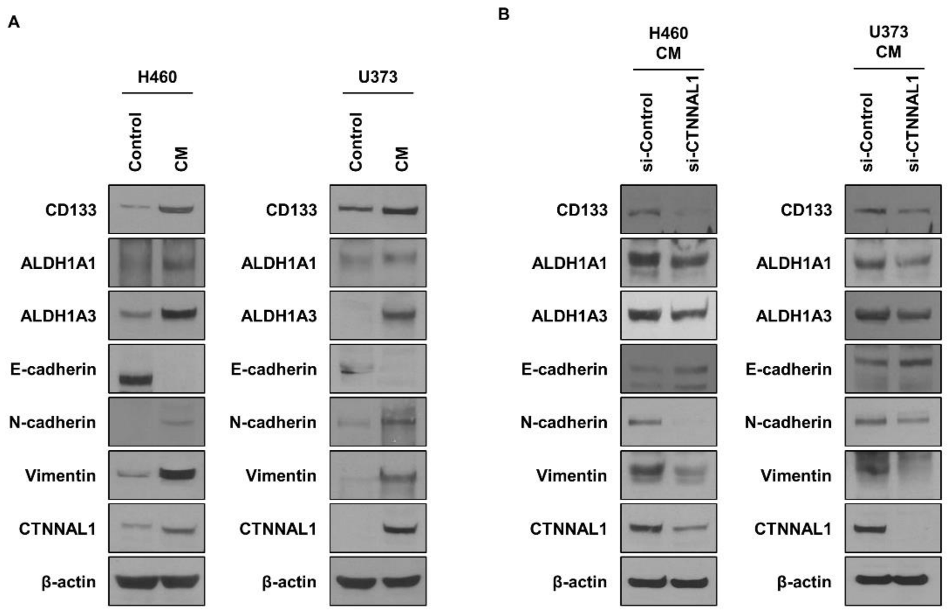

Supplementary Figure S1. Regulation of CSC and EMT marker proteins by CTNNAL1 under CM conditions. (A) Comparison of the expression of CSC and EMT marker proteins in H460 lung cancer cells and U373 glioma cells under CM conditions. (B) Comparison of the expression of CSC and EMT marker proteins when CTNNAL1 was inhibited by treating H460 and U373 cells with si-RNA.

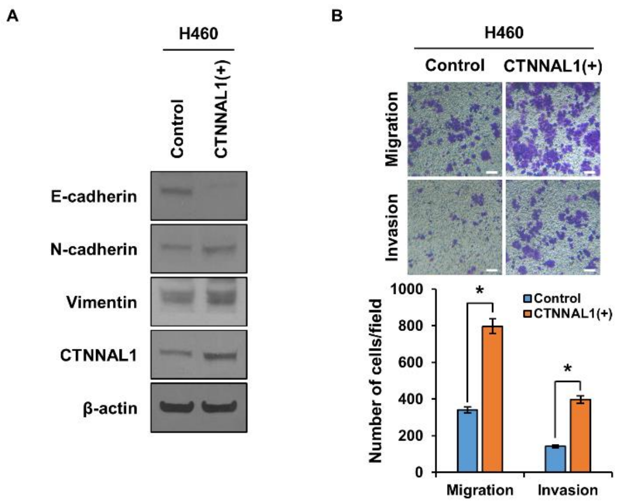

Supplementary Figure S2. Observation of EMT phenomenon in H460 cells overexpressing CTNNAL1. (A) Identification of EMT marker proteins after overexpressing CTNNAL1 in H460 cells. (B) Measurement of migration and invasion ability of H460 cells overexpressing CTNNAL1. Error bars represent mean ± SD of triplicate samples. *p<0.001 versus control. Scale bar = 50 μm
